# Predictive Factors of Early Recurrence in Patients with Distal Cholangiocarcinoma after Pancreaticoduodenectomy

**DOI:** 10.1155/2018/6431254

**Published:** 2018-04-03

**Authors:** Yasuhiro Ito, Yuta Abe, Tomohisa Egawa, Minoru Kitago, Osamu Itano, Yuko Kitagawa

**Affiliations:** ^1^Department of Surgery, Saiseikai Yokohamashi Tobu Hospital, 3-6-1 Shimosueyoshi, Tsurumi-ku, Yokohama-shi, Kanagawa 230-0012, Japan; ^2^Department of Surgery, Keio University School of Medicine, 35 Shinanomachi, Shinjuku-ku, Tokyo 160-8582, Japan

## Abstract

**Aim:**

To determine the factors associated with early recurrence in patients with distal cholangiocarcinoma after pancreaticoduodenectomy (PD).

**Patients and Methods:**

Sixty-one patients with distal cholangiocarcinoma were enrolled. The clinical data and histopathological findings were collected retrospectively.

**Results:**

Patients were divided into two groups as follows: 16 patients (26%) with early recurrence and 45 patients (74%) with late recurrence or no recurrence. In a univariate analysis, lymph node metastases (*P* = 0.0016), lymphatic invasion (*P* < 0.0001), pancreatic invasion (*P* = 0.0006), and perineural invasion (*P* = 0.0004) were significantly different between the two groups. In a multivariate analysis, a higher incidence of lymphatic invasion was the only independent risk factor for early recurrence (odds ratio: 5.772, 95% confidence interval: 1.123–29.682, *P* = 0.036). Moreover, the disease-free survival and overall survival of patients with a higher incidence of lymphatic invasion were significantly worse compared with those of patients with a lower incidence of lymphatic invasion (*P* < 0.001).

**Conclusions:**

Our study showed that a higher incidence of lymphatic invasion was a significant predictor of early recurrence in patients with distal cholangiocarcinoma. Therefore, lymphatic invasion might be useful in determining the optimal adjuvant therapy in the early postoperative stage for distal cholangiocarcinoma.

## 1. Introduction

It is difficult to diagnose cholangiocarcinoma in the early stages because most patients present with jaundice, which is generally thought to be the most important symptom at diagnosis. Because these tumors are likely to involve the surrounding vessels and nerves, vessel involvement indicates unresectability and neural invasion can suggest a poor prognosis. Extensive surgery remains the only curative treatment for these tumors.

The 5-year overall and R0 survival rates for patients with bile duct cancer were 18% and 30%, respectively, and the median survival was 15 and 28 months, respectively. For patients with intrahepatic, perihilar, and distal tumors, the 5-year survival rate was 40%, 10%, and 23%, respectively, and the median survival was 28, 13, and 18 months, respectively [1]. Despite improvements in surgical instruments and techniques, the prognosis is not yet satisfactory because of the high incidence of local or regional recurrence and distant metastasis. Because there are few studies of early recurrence in patients with distal cholangiocarcinoma after surgical resection, the prognosis remains unclear. However, clinicians assume that early recurrence affects overall survival. Additionally, they recognize that additional treatment after diagnosis of early recurrence might be insufficient.

There are limitations to detecting occult tumors, such as micrometastasis, at the time of surgery. Therefore, adjuvant treatment is expected, even though there is no established adjuvant therapy for distal cholangiocarcinoma. Investigating the predictive factors of early recurrence might prolong survival by optimizing adjuvant therapy soon after surgery.

The aim of this study was to determine the factors associated with early recurrence after surgical resection in patients with distal cholangiocarcinoma.

## 2. Patients and Methods

### 2.1. Protocol

Between April 2005 and April 2015, 61 patients with distal cholangiocarcinoma underwent curative resection at our institution. The Clinical Ethics Committee of Saiseikai Yokohamashi Tobu Hospital approved this study. All patients were histologically diagnosed with distal cholangiocarcinoma. All the operations were performed by experienced pancreatic surgeons. Lymph nodes were dissected routinely. The lymph nodes around the head of the pancreas (LN 13, LN 17), the common hepatic artery (LN 8), the superior mesenteric artery (LN 14), and the hepatoduodenal ligament (LN 12) were dissected during pancreatectomy [2]. The neural plexus around the superior mesenteric artery was not dissected. All soft tissues around them were completely dissected and skeletonized. After resection, reconstructions were performed according to the modified Child method or Traverso method.

### 2.2. Data Collection

The preoperative demographic and clinical data, surgical procedure, and pathologic diagnosis details were collected retrospectively. The histopathological factors were classified according to the Japanese Society of Biliary Surgery [2] and included tumor size, differentiation (papillary, well, moderately, or poorly), status of invasion (lymphatic, venous, perineural, pancreatic, or duodenal), and surgical margin status. Depth of invasion into the bile duct wall and lymph node involvement were defined according to the tumor-node-metastasis (TNM) classification, seventh edition [3]. The degree of lymphatic invasion, venous invasion, perineural invasion, pancreatic invasion, and duodenal invasion was classified in detail as follows: ly0, v0, pn0, panc0, and du0 = no evidence of invasion; ly1, v1, pn1, panc1, and du1 = mild invasion; ly2, v2, pn2, panc2, and du2 = moderate invasion; and ly3, v3, pn3, panc3, and du3 = severe invasion, respectively [2]. Based on this result, furthermore, pathologic factors were divided into two categories (lower incidence (<2) and higher incidence (≥2)).

Patients received follow-up with laboratory tests including tumor marker measurements and ultrasonography or computed tomography every 3 months during the first 3 years. If patients had no evidence of recurrence at 3 years after surgery, they were followed up at 6-month intervals. No adjuvant chemotherapy was administered to any of the patients. Recurrence was confirmed by radiological examination or elevation of tumor markers. Early and late recurrences were defined as occurring within 1 year and after 1 year, respectively. Patients who were followed up with no evidence of recurrence within 1 year after surgery were excluded to avoid the possibility of recurrence occurring thereafter.

### 2.3. Statistical Analysis

Continuous data are expressed as the mean ± standard deviation (SD). The chi-square test or Fisher's exact test was used to compare categorical data, and Student's *t*-test or the Mann–Whitney *U* test was used for continuous data, as appropriate. Logistic regression was performed for a multivariate analysis to determine the independent risk factors. The disease-free and overall survival curves were estimated using the Kaplan-Meier method and compared by the log-rank test. A *P* value < 0.05 was considered statistically significant. Statistical analyses were done using SPSS 19.0 software (SPSS Japan Inc., Tokyo, Japan).

## 3. Results

From April 2005 to April 2015, a total of 61 consecutive patients with distal cholangiocarcinoma underwent surgical treatment. They comprised 41 men and 20 women, with an average age of 69.5 years (range, 50–84). Forty-six of the 61 patients (75%) had jaundice. All of them and 9 patients who did not have jaundice underwent biliary drainage: endoscopic retrograde biliary drainage in 50 and percutaneous transhepatic biliary drainage in 5. The type of operation was as follows: pancreaticoduodenectomy (PD) in 5 (8.2%), pylorus-preserving PD (PPPD) in 20 (32.8%), and subtotal stomach-preserving PD (SSPPD) in 36 (59.0%). The tumor stage according to the TNM classification was as follows: stage IA in 25 (41.0%), IB in 14 (23.0%), IIA in 3 (4.9%), and IIB in 19 (31.1%). There were 18.52 ± 9.34 lymph nodes and 0.51 ± 0.99 involved lymph nodes. The characteristics of all patients are listed in Table 1. The disease-free 1-, 3-, and 5-year survival rates were 75.4%, 65.0%, and 59.0%, respectively. The overall 1-, 3-, and 5-year survival rates were 86.9%, 68.9%, and 66.4%, respectively.

**Figure 1:**
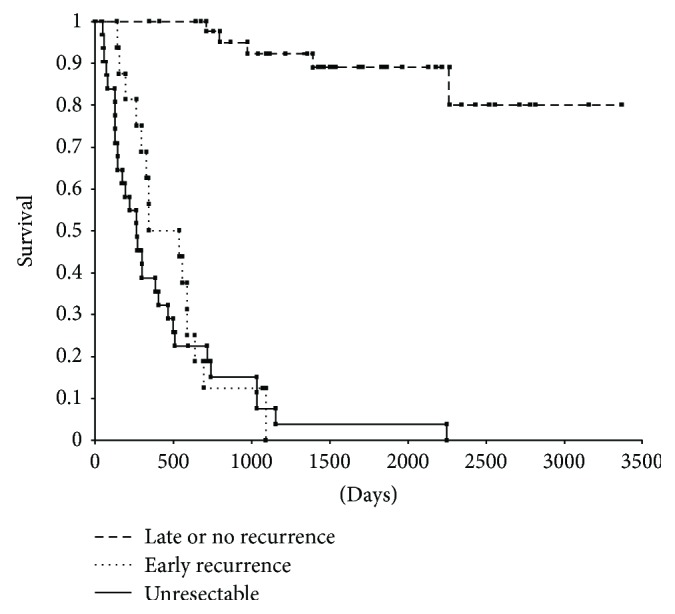
Overall survival for 3 groups (unresectable (*n* = 33), early recurrence (*n* = 16), and late and no recurrence (*n* = 45)).

Of all the patients, 24 patients had recurrences, and the sites of initial recurrence were the lymph nodes in 10 patients (42%), liver in 9 (38%), local in 8 (33%), peritoneum in 7 (29%), and lungs in 4 (17%). Furthermore, all patients were divided into three groups as follows: 16 patients (26%) with early recurrence, 8 patients (13%) with late recurrence, and 37 patients (61%) with no recurrence. There was no significant difference in the pattern of the recurrence site according to the timing of recurrence (Table 2). Thirty-three patients who did not undergo resection during the same period in this study were categorized as an unresectable group at the multidisciplinary team meeting. They comprised 20 men and 13 women, with an average age of 77.8 (range, 56–91) years. Twenty-eight of the 33 patients (85%) had jaundice. Thirty patients (91%) underwent biliary drainage. The reasons for unresectability were as follows: distant metastases in 16 (48%), locally advanced tumor in 7 (21%), peritoneal location in 3 (9%), and other reasons in 7 (7%). Although the operation was attempted in 4 patients, they were unexpectedly diagnosed as unresectable. They were compared with patients with early recurrence and patients without early recurrence (late and no recurrence). The overall survival time was significantly different between patients with early recurrence and patients without early recurrence (*P* < 0.001), but there was no significant difference between patients with early recurrence and patients who did not undergo resection (*P* = 0.5688) (Figure 1).

There were 16 patients in the early recurrence group and 45 patients in the combined late recurrence plus no recurrence group. The demographic, perioperative, and pathological factors were compared between these two groups. In a univariate analysis, lymph node metastases (*P* = 0.002), lymphatic invasion (*P* < 0.001), pancreatic invasion (*P* = 0.001), and perineural invasion (*P* < 0.001) were significantly different between the two groups (Table 3). On the other hand, there were no significant differences in gender, age, jaundice, preoperative biliary drainage, serum CA19-9 value, operative time, intraoperative bleeding, blood transfusion, type of operation, pancreatic fistula, delayed gastric emptying, tumor differentiation, venous invasion, duodenal invasion, and R status. There was no significant difference in the number of lymph nodes between the two groups (data not shown). Therefore, the extent of lymph node dissection was almost equivalent. In a multivariate analysis, a higher incidence of lymphatic invasion was the only independent risk factor for early recurrence (odds ratio [OR]: 5.77, 95% confidence interval [CI]: 1.12–29.68, *P* = 0.036) (Table 3).

**Figure 2:**
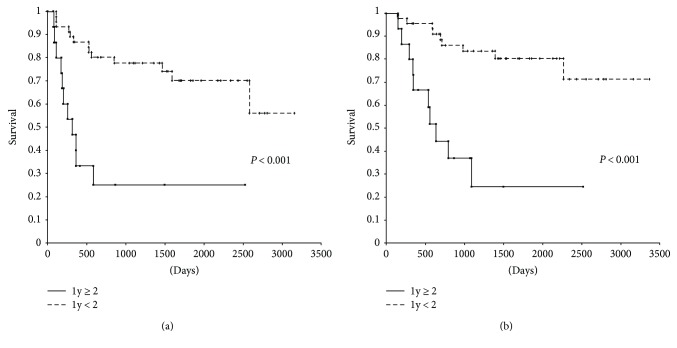
Disease-free survival and overall survival for two categories (lower incidence (ly < 2) and higher incidence (ly ≥ 2)).

Disease-free survival of patients with a higher incidence of lymphatic invasion (ly ≥ 2) was significantly worse compared with that of patients with a lower incidence of lymphatic invasion (ly < 2) (*P* < 0.001) (Figure 2(a)). Similarly, overall survival of patients with a higher incidence of lymphatic invasion (ly ≥ 2) was significantly worse compared with that of patients with a lower incidence of lymphatic invasion (ly < 2) (*P* < 0.001) (Figure 2(b)). In patients with a lower incidence of lymphatic invasion, the disease-free 1-, 3-, and 5-year survival rates and the overall 1-, 3-, and 5-year survival rates were 87.0%, 77.8%, and 70.2% and 95.7%, 83.6%, and 80.4%, respectively. In patients with a higher incidence of lymphatic invasion, the disease-free 1-, 3-, and 5-year survival rates and the overall 1-, 3-, and 5-year survival rates were 46.7%, 11.6%, and 11.6% and 66.7%, 24.7%, and 24.7%, respectively.

## 4. Discussion

Many studies of prognostic factors for distal cholangiocarcinoma have been reported, such as lymph node metastases [4, 5], surgical margin [6, 7], tumor differentiation [8, 9], T factor [10, 11], lymphatic invasion [12, 13], venous invasion [14, 15], pancreatic invasion [16, 17], and perineural invasion [15, 18]. To date, the prognostic factors have not yet been defined. Kiriyama et al. [5] reported that the total lymph node count (TLNC) significantly affected survival and that a large number of TLNC could prevent the migration of involved nodes. Similarly, they reported that the number of involved nodes was a significant prognostic factor.

In this study, we showed that the overall survival time was not significantly different between patients with early recurrence and patients who did not undergo resection (*P* = 0.5688). Analyzing patients with early recurrence might help to avoid unnecessary surgery or to suggest the necessity of adjuvant therapy. Several reports have been published concerning the recurrence of distal cholangiocarcinoma. Woo et al. [19] reported that lymph node involvement was the only significant factor of recurrence in extrahepatic distal cholangiocarcinoma. Lymph node involvement is considered a risk factor for recurrence with a similar survival rate and such a poor prognostic factor that an operation might not be indicated at all. Clinicians mostly depend on pathologic findings for an analysis of recurrence and survival; therefore, there is an incentive to predict lymph node involvement preoperatively. Noji et al. reported that a computed tomography (CT) scan is not ineffective for assessing surgical indication based on paraaortic lymph node size and morphology in patients with biliary carcinoma [20]. A previous study reported that fluorodeoxyglucose positron emission tomography (FDG-PET) was useful for predicting lymph node metastasis [21], and lymph node metastasis detected on 18F-FDG PET/CT had a positive correlation with 1-year recurrence after surgical resection in patients with peripheral intrahepatic cholangiocarcinoma [22]. It may be useful for diagnosing lymph node involvement preoperatively and avoiding unnecessary operation. Fouquet et al. [23] reported that perineural invasion was a predictor of early recurrence in patients with pancreatic head adenocarcinoma. Woo et al. [19] identified venous invasion and perineural invasion as risk factors for recurrence of ampullary carcinoma after radical resection. However, very few studies have focused on the early recurrence of distal cholangiocarcinoma. To our knowledge, our study is the first to evaluate the early recurrence of distal cholangiocarcinoma.

Our study showed that a higher incidence of lymphatic invasion is an independent predictor of early recurrence, and the disease-free survival and overall survival of patients with a higher incidence of lymphatic invasion were significantly worse than those of patients with a lower incidence of lymphatic invasion (*P* < 0.001). Therefore, we hypothesize that the survival of patients with a higher incidence of lymphatic invasion might be beneficially influenced by adjuvant chemotherapy at an early stage after curative surgery. Of 32 patients with lymphatic invasion (ly > 1), 17 patients (53.1%) had lymph node involvement, and this was similar to the higher incidence of lymphatic invasion (9/15; 60%). Hence, we suggest that lymphatic invasion is strongly associated with lymph node involvement. In addition, Aoyama et al. [24] reported that lymphatic invasion was associated with liver metastasis in pancreatic cancer patients. In our study, of 32 patients with lymphatic invasion (ly > 1), 9 patients (28.1%) had liver metastasis, compared with none of 29 patients without lymphatic invasion (*P* = 0.002). Our results support those of a previous report of the correlation between liver metastasis and lymphatic invasion; therefore, lymphatic invasion must be considered a precursor of occult tumors. Considering these results, lymphatic invasion has the potential to be a predictor of early recurrence after curative resection, and those with lymphatic invasion might benefit from adjuvant therapy.

For resectable cholangiocarcinoma, curative surgery is vital. Because, however, the ability of surgery to improve the recurrence and survival rates is limited, we expect to treat patients with adjuvant therapy, similar to the treatment for other gastrointestinal cancers [25–29]. A retrospective study from a single institution that compared results to those of a historical series showed that adjuvant gemcitabine plus S-1 chemotherapy improved 5-year survival (*P* < 0.001) [30]. The multivariable analysis revealed that the use of postoperative adjuvant chemotherapy (hazard ratio [HR] = 2.82, 95% CI: 1.53–5.22, *P* < 0.001) and surgical margin (HR = 2.49, 95% CI: 1.37–4.52, *P* = 0.003) are independent prognostic factors. A randomized controlled study reported postoperative adjuvant chemotherapy for pancreaticobiliary carcinoma [31]. The 5-year survival rate of patients with gallbladder carcinoma in a chemotherapy group that received mitomycin C and 5-fluorouracil after surgery was significantly different from that of the surgery-alone group (*P* = 0.0367). In addition, classifying the type of curability as curative or noncurative, we showed that the 5-year survival rate of patients with gallbladder carcinoma after noncurative resection was significantly different from that of the surgery-alone group (*P* = 0.0226). However, there was no significant difference in survival of patients with other types of carcinoma (pancreas, bile duct, and ampulla of Vater) between curative and noncurative resection. Therefore, the efficiency of postoperative adjuvant chemotherapy for pancreaticobiliary carcinoma without gallbladder carcinoma after noncurative resection was not shown. In a randomized controlled trial for periampullary cancer, the European Study Group of Pancreatic Cancer (ESPAC-3) showed that there was no significant difference between the chemotherapy (gemcitabine and fluorouracil + folinic acid) group and the observation group (HR = 0.86, 95% CI: 0.66–1.11, *P* = 0.25) [32]. On the other hand, a multivariable analysis revealed that chemotherapy was an independent prognostic factor (HR = 0.75, 95% CI: 0.57–0.98, *P* = 0.03). In addition, the BILCAP multicenter prospective, randomized controlled phase III trial in the United Kingdom is being conducted to define the role of adjuvant chemotherapy—oral fluoropyrimidine (capecitabine)—for biliary tract cancer after curative surgical resection [33]. Adjuvant chemotherapy with capecitabine in biliary tract cancer has been shown to improve overall survival. This multidisciplinary management may be a valid option to help improve overall survival and will become a standard of care. Therefore, attention must be paid to these results in the future.

In our study, a higher incidence of lymphatic invasion was an independent factor for early recurrence. Therefore, administering the optimal adjuvant therapy for distal cholangiocarcinoma might be significantly beneficial. However, our study is limited because the number of patients is small and it is a retrospective, nonrandomized study. To our knowledge, there is currently no established treatment strategy after surgery for distal cholangiocarcinoma. A study with a large number of patients with distal cholangiocarcinoma is required to elucidate the risk factors for early recurrence. Additionally, a well-designed randomized controlled trial is needed to determine the survival benefit by adjuvant therapy.

## 5. Conclusion

Many studies have reported that the presence of lymph node involvement is a significant prognostic factor after curative resection of distal cholangiocarcinoma. However, predictors of early recurrence after resection for distal cholangiocarcinoma remain unclear, because few studies have focused on early recurrence in resectable distal cholangiocarcinoma. Recognizing the predictive factors of early recurrence, which cannot be detected preoperatively, we might confer an additional survival benefit by allowing for treatment adjustments in the early postoperative stage. Our study showed that a higher incidence of lymphatic invasion was associated with early recurrence with poor disease-free survival and overall survival. Thus, it is a useful predictor of early recurrence after surgical resection for distal cholangiocarcinoma.

## Figures and Tables

**Table 1:** Characteristics in patients who underwent pancreaticoduodenectomy (*n* = 61).

Gender (male/female)	41/20
Age (mean ± SD)	69.49 ± 8.99
BMI (kg/m^2^)	22.6 ± 3.29
Jaundice (yes/no)	
Yes	46 (75%)
No	15 (25%)
Preoperative biliary drainage (yes/no)	
Yes	55 (90%)
No	6 (10%)
TNM stage	
IA	25 (41%)
IB	14 (23%)
IIA	3 (5%)
IIB	19 (31%)
Type of operation	
PD	5 (8%)
SSPPD	20 (33%)
PPPD	36 (59%)
Lymph node	
A number of lymph nodes	18.52 ± 9.34
A number of involved lymph nodes	0.51 ± 0.99
R status	
R0	57 (93%)
R1	4 (7%)
R2	0

SD: standard deviation; BMI: body mass index; PD: pancreaticoduodenectomy; SSPPD: subtotal stomach-preserving pancreaticoduodenectomy; PPPD: pylorus-preserving pancreaticoduodenectomy.

**Table 2:** Location of the first recurrence site.

	Early recurrence (*n* = 16)	Late recurrence (*n* = 8)	*P* value
Lymph node	8 (50%)	2 (25%)	0.388
Liver	6 (38%)	3 (38%)	0.999
Local	5 (31%)	3 (38%)	0.999
Peritoneum	6 (38%)	1 (13%)	0.352
Lung	2 (13%)	2 (25%)	0.578

**Table 3:** Univariate and multivariate analyses of risk factors for postoperative early recurrence.

		Univariate			Multivariate		
		Early recurrence (*n* = 16)	Late or no recurrence (*n* = 45)	*P* value	Odds ratio	95% CI	*P* value
Gender	Male	12	29	0.544			
	Female	4	16				

Age (yr)	≥70	11	21	0.129			
	<70	5	24				

Jaundice	Yes	14	32	0.312			
	No	2	13				

Preoperative biliary drainage	Yes	15	40	0.999			
	No	1	5				

CA19-9 (U/ml)	≤100	9	34	0.146			
	>100	7	11				

Operative time (min)	≥420	12	22	0.071			
	<420	4	23				

Bleeding (ml)	≤1000	8	25	0.702			
	>1000	8	20				

Transfusion	Yes	6	12	0.415			
	No	10	33				

Type of operation	PD	3	2	0.139			
	SSPPD	6	14				
	PPPD	7	29				

Pancreatic fistula	Yes	8	23	0.939			
	No	8	22				

Delayed gastric emptying	Yes	1	2	0.999			
	No	15	43				

Lymph node metastases	Positive	10	9	0.002	3	0.66–13.44	0.158
	Negative	6	36				

Differentiation	Papillary	1	5	0.059			
	Well	6	31				
	Moderately	7	7				
	Poorly	2	2				

Lymphatic invasion	ly < 2	6	40	<0.001	5.77	1.12–29.68	0.036
	ly ≥ 2	10	5				

Venous invasion	v < 2	16	41	0.565			
	v ≥ 2	0	4				

Pancreatic invasion	panc < 2	7	39	<0.001	1.41	0.23–8.79	0.713
	panc ≥ 2	9	6				

Perineural invasion	pn < 2	5	36	<0.001	2.78	0.53–14.5	0.226
	pn ≥ 2	11	9				

Duodenal invasion	du < 2	14	41	0.648			
	du ≥ 2	2	4				

R status	R0	13	44	0.052			
	R1	3	1				

CI: confidence interval.

## References

[1] DeOliveira M. L., Cunningham S. C., Cameron J. L. (2007). Cholangiocarcinoma: thirty-one-year experience with 564 patients at a single institution. *Annals of Surgery*.

[2] Japanese Society of Biliary Surgery (2004). *Classification of Biliary Tract Carcinoma*.

[3] Sobin L. H., Gospodarowicz M. K., Wittekind C. (2009). *TNM Classification of Malignant Tumours. International Union Against Cancer (UICC)*.

[4] Tan X., Xiao K., Liu W., Chang S., Zhang T., Tang H. (2013). Prognostic factors of distal cholangiocarcinoma after curative surgery: a series of 84 cases. *Hepato-Gastroenterology*.

[5] Kiriyama M., Ebata T., Aoba T. (2015). Prognostic impact of lymph node metastasis in distal cholangiocarcinoma. *British Journal of Surgery*.

[6] Murakami Y., Uemura K., Hayashidani Y. (2007). Prognostic significance of lymph node metastasis and surgical margin status for distal cholangiocarcinoma. *Journal of Surgical Oncology*.

[7] Qiao Q. L., Zhang T. P., Guo J. C. (2011). Prognostic factors after pancreatoduodenectomy for distal bile duct cancer. *The American Surgeon*.

[8] Wakai T., Shirai Y., Moroda T., Yokoyama N., Hatakeyama K. (2005). Impact of ductal resection margin status on long-term survival in patients undergoing resection for extrahepatic cholangiocarcinoma. *Cancer*.

[9] Argani P., Shaukat A., Kaushal M. (2001). Differing rates of loss of *DPC4* expression and of p53 overexpression among carcinomas of the proximal and distal bile ducts. *Cancer*.

[10] Ebata T., Nagino M., Nishio H., Igami T., Yokoyama Y., Nimura Y. (2007). Pancreatic and duodenal invasion in distal bile duct cancer: paradox in the tumor classification of the American Joint Committee on Cancer. *World Journal of Surgery*.

[11] Chung Y. J., Choi D. W., Choi S. H., Heo J. S., Kim D. H. (2013). Prognostic factors following surgical resection of distal bile duct cancer. *Journal of the Korean Surgical Society*.

[12] Fisher S. B., Patel S. H., Kooby D. A. (2012). Lymphovascular and perineural invasion as selection criteria for adjuvant therapy in intrahepatic cholangiocarcinoma: a multi-institution analysis. *HPB*.

[13] Kwon H. J., Kim S. G., Chun J. M., Lee W. K., Hwang Y. J. (2014). Prognostic factors in patients with middle and distal bile duct cancers. *World Journal of Gastroenterology*.

[14] Takao S., Shinchi H., Uchikura K., Kubo M., Aikou T. (1999). Liver metastases after curative resection in patients with distal bile duct cancer. *British Journal of Surgery*.

[15] Hong S. M., Pawlik T. M., Cho H. (2009). Depth of tumor invasion better predicts prognosis than the current American Joint Committee on Cancer T classification for distal bile duct carcinoma. *Surgery*.

[16] Cheng Q., Luo X., Zhang B., Jiang X., Yi B., Wu M. (2007). Distal bile duct carcinoma: prognostic factors after curative surgery. A series of 112 cases. *Annals of Surgical Oncology*.

[17] Choi S. B., Park S. W., Kim K. S., Choi J. S., Lee W. J. (2009). The survival outcome and prognostic factors for middle and distal bile duct cancer following surgical resection. *Journal of Surgical Oncology*.

[18] Kawai M., Tani M., Kobayashi Y. (2010). The ratio between metastatic and examined lymph nodes is an independent prognostic factor for patients with resectable middle and distal bile duct carcinoma. *The American Journal of Surgery*.

[19] Woo S. M., Ryu J. K., Lee S. H. (2007). Recurrence and prognostic factors of ampullary carcinoma after radical resection: comparison with distal extrahepatic cholangiocarcinoma. *Annals of Surgical Oncology*.

[20] Noji T., Kondo S., Hirano S. (2005). CT evaluation of paraaortic lymph node metastasis in patients with biliary cancer. *Journal of Gastroenterology*.

[21] Seo S., Hatano E., Higashi T. (2008). Fluorine-18 fluorodeoxyglucose positron emission tomography predicts lymph node metastasis, P-glycoprotein expression, and recurrence after resection in mass-forming intrahepatic cholangiocarcinoma. *Surgery*.

[22] Park T. G., Yu Y. D., Park B. J. (2014). Implication of lymph node metastasis detected on 18F-FDG PET/CT for surgical planning in patients with peripheral intrahepatic cholangiocarcinoma. *Clinical Nuclear Medicine*.

[23] Fouquet T., Germain A., Brunaud L., Bresler L., Ayav A. (2014). Is perineural invasion more accurate than other factors to predict early recurrence after pancreatoduodenectomy for pancreatic head adenocarcinoma?. *World Journal of Surgery*.

[24] Aoyama T., Murakawa M., Katayama Y. (2015). Lymphatic invasion is an independent prognostic factor in pancreatic cancer patients undergoing curative resection followed by adjuvant chemotherapy with gemcitabine or S-1. *Hepato-Gastroenterology*.

[25] Macdonald J. S., Smalley S. R., Benedetti J. (2001). Chemoradiotherapy after surgery compared with surgery alone for adenocarcinoma of the stomach or gastroesophageal junction. *The New England Journal of Medicine*.

[26] Sakuramoto S., Sasako M., Yamaguchi T. (2007). Adjuvant chemotherapy for gastric cancer with S-1, an oral fluoropyrimidine. *The New England Journal of Medicine*.

[27] Wolmark N., Rockette H., Fisher B. (1993). The benefit of leucovorin-modulated fluorouracil as postoperative adjuvant therapy for primary colon cancer: results from National Surgical Adjuvant Breast and Bowel Project Protocol C-03. *Journal of Clinical Oncology*.

[28] Lembersky B. C., Wieand H. S., Petrelli N. J. (2006). Oral uracil and tegafur plus leucovorin compared with intravenous fluorouracil and leucovorin in stage II and III carcinoma of the colon: results from National Surgical Adjuvant Breast and Bowel Project Protocol C-06. *Journal of Clinical Oncology*.

[29] Twelves C., Wong A., Nowacki M. P. (2005). Capecitabine as adjuvant treatment for stage III colon cancer. *The New England Journal of Medicine*.

[30] Murakami Y., Uemura K., Sudo T. (2009). Adjuvant gemcitabine plus S-1 chemotherapy improves survival after aggressive surgical resection for advanced biliary carcinoma. *Annals of Surgery*.

[31] Study Group of Surgical Adjuvant Therapy for Carcinomas of the Pancreas and Biliary Tract, Takada T., Amano H. (2002). Is postoperative adjuvant chemotherapy useful for gallbladder carcinoma? A phase III multicenter prospective randomized controlled trial in patients with resected pancreaticobiliary carcinoma. *Cancer*.

[32] Neoptolemos J. P., Moore M. J., Cox T. F. (2012). Effect of adjuvant chemotherapy with fluorouracil plus folinic acid or gemcitabine vs observation on survival in patients with resected periampullary adenocarcinoma: the ESPAC-3 periampullary cancer randomized trial. *JAMA*.

[33] Primrose J. N., Fox R. P., Palmer D. H. (2017). Adjuvant capecitabine for biliary tract cancer: the BILCAP randomized study. *Journal of Clinical Oncology*.

